# Lingual Taste Neuroepithelium Precursor Hippocampal Implantation: A Novel Neurosurgical Intervention Hypothesis for Alzheimer’s Dementia

**DOI:** 10.1002/alz.095395

**Published:** 2025-01-09

**Authors:** Youssef A. Ismail, Hazim Mohamed, Youssef M. Abd El‐Satar, Youssef Haitham, Mohammad Walid

**Affiliations:** ^1^ Faculty of Medicine Port Said Univeristy, Egypt, Port Said, Port Said Egypt; ^2^ Faculty of Medicine Helwan University, Cairo, Helwan Egypt; ^3^ Faculty of Medicine Cairo Univeristy, Egypt, Cairo, Cairo Egypt; ^4^ Faculty of Medicine Suez Canal Univeristy, Egypt, Ismailia, Ismailia Egypt; ^5^ Faculty of Medicine, Arish University, Arish, North Sinai Egypt

## Abstract

**Background:**

Lingual taste cells (LTCs) are taste buds' sensory cells that modulate gustation. This study’s aim is to assess whether it can be successfully implanted in hippocampus, modulating learning and memory deficits observed in Alzheimer’s Dementia (AD).

**Methods:**

Retrospective trials on rodents i.e., rats and guinea pigs showed that LTCs have been detected to demonstrate similarities with neurons, releasing neurotransmitters, synapsing and even antagonizing pathologies associated with AD. We mapped how it possibly can enhance cognitive function and memory deficits. Previous literature will be demonstrated in this study supporting and/or opposing therapeutic perspective.

**Results:**

LTCs are classified into type I (glial‐like), type II receptor, and type III presynaptic cells. Preliminary evaluation noted that only type III LTCs possess conventional synapses, and transmission of taste stimulus from type II to nerve fiber requires a non‐conventional pathway, by subsurface cisternae and specialized mitochondria and also suggesting neurotransmitters. Type II LTCs have been identified to secrete acetylcholine, and type III’s have also serotonin (5‐HT), γ‐aminobutyric acid (GABA), and noradrenalin. Type II LTCs have been also found to express taste receptor type 1 member 1 (T1R_1_) and 3 (T1R_3_), been involved in the detection of glutamate gustatory stimulus from afferent nerve fibers. In long‐withstanding AD patients, decreased intracellular and increased extracellular adenosine triphosphate (ATP), by ATP synthase deficiency and P2X_7_ respectively, have been evident. Purinergic signaling in LTCs is also evident, type II has been recognized to secrete ATP by Panx1 with taste stimulation, and hydrolyzed by nucleoside triphosphate diphosphohydrolase‐2 (NTPDase2), expressed on the membranes of type I LTCs, which constitute the majority of cells within each bud. ATP also activates sour‐sensitive type III LTCs via P2Y_4_, causing vesicular release of 5‐HT and GABA, which in turn induce negative feedback on ATP release from type II LTCs. Glutamate also activates ionotropic glutamate receptors, N‐methyl‐D‐aspartate receptor (NMDAR), in type III LTCs, secreting 5‐HT and inducing previous feedback.

**Conclusion:**

LTCs possess set of characteristics that could possibly compensating learning and memory impairment in AD, even in neuroplasticity in autism spectrum disorder (ASD), multiple sclerosis (MS) and other neurodegenerative diseases.

